# Multicolor and Attenuated Light Intensity Responses in Protein Hydrogels Arising from Photoregulated Crosslink Dynamics

**DOI:** 10.1002/anie.6692301

**Published:** 2026-05-28

**Authors:** Saskia Frank, Seraphine V. Wegner

**Affiliations:** ^1^ Institute of Physiological Chemistry and Pathobiochemistry University of Münster Münster Germany

**Keywords:** attenuated response, Cph1, multicolor, photoresponsive, protein hydrogel

## Abstract

Light‐responsive hydrogels enable noninvasive, precise, remote control over material properties with great biocompatibility, yet achieving multicolor addressability and dynamic regulation of mechanical properties remains challenging. Most systems rely on single photoswitches and regulate stiffness primarily through changes in crosslinking density. Here, we present a fully protein‐based hydrogel that combines multicolor light responsiveness with optical control of crosslink dynamics. The hydrogel includes two visible light‐responsive photoceptors: the cyanobacterial phytochrome Cph1, which enables reversible red/far‐red light‐controlled crosslinking, and CarH, which introduces irreversible green light‐induced gel‐sol transitions. Remarkably, Cph1‐based hydrogels exhibited an attenuated red light intensity response, forming stiffer networks under low‐intensity illumination than under high‐intensity light and show a subsequent dark‐adaptation with stiffening once red light illumination is stopped. This counterintuitive behavior arises from light‐driven bidirectional photoisomerization that modulates crosslink lifetimes without altering the photostationary state composition. Together, these findings establish orthogonally addressable reversible and irreversible crosslinks and photoregulated crosslink dynamics as new design principles for multicolor light‐responsive biomaterials.

## Graphical abstract

A fully protein‐based hydrogel integrated two orthogonal photoreceptors to enable reversible manipulation of stiffness with red/far‐red light and irreversible gel‐sol transitions with green light. Unexpectedly, high‐intensity red light results in attenuated stiffening and dark‐adaptation, arising from increased crosslinking dynamics in the photostationary state, a new design principle for light‐responsive biomaterials.

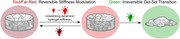

## 1 Introduction

Hydrogels are central to biomedical materials research due to their high biocompatibility and tissue‐like mechanical properties. Introducing responsiveness to stimuli, including pH, temperature, chemical signals or light into hydrogels enables dynamic control over structure and mechanics, thereby better mimicking the adaptive nature of biological materials [1, 2, 3, 4, 5]. Among external triggers, light is uniquely powerful, as it allows remote, noninvasive actuation with great tunability of illumination parameters (e.g., intensity, pulsing) and superb spatial and temporal precision. In particular, visible light enables selective activation of defined molecular processes while maintaining excellent biocompatibility. Light‐responsive hydrogels have been realized using a variety of light‐responsive building blocks. Hydrogels with irreversible light responses rely on photocleavable [2, 6, 7, 8, 9, 10] and photoactivatable covalent crosslinks [10, 11, 12, 13, 14], whereas reversible changes often employ photoswitches such as azobenzenes [15, 16, 17] or arylazopyrazoles [18] combined with supramolecular guest‐host interactions with cyclodextrins [19, 20, 21], pseudopolyrotaxane [22], or cucurbit[n]urils [23, 24]. To avoid UV light irradiation, which can compromise biocompatibility [25, 26], visible light‐responsive protein photoreceptors including PhoCl [2, 7], Cph1 [5, 27, 28], LOVTRAP [29], CarH [4, 30], PYP [31, 32], and Dronpa145N [3, 33] have been incorporated into hydrogels. In some cases, this has yielded fully protein‐based materials, eliminating the need for chemical conjugation of the photoresponsive protein to a synthetic polymer backbone [3, 4, 29, 30, 34]. Light‐responsive hydrogels are particularly powerful tools for studying cell‐matrix interactions, as the extracellular matrix (ECM) plays a central role in regulating cell behavior and fate [35, 36, 37]. Dynamic, light‐tuneable hydrogels have been used to investigate mechanoresponses during cell differentiation [5, 31], cell migration [33], control cell release from materials [3, 34], spatially resolve exposure to immobilized factors [38, 39], and regulate organoid and tissue development [40, 41]. Considering the multitude of biological processes, mechanically tuneable hydrogels that respond to multiple orthogonal wavelengths are especially promising, with reported examples including (UV‐) visible light‐induced degradation [8, 42], photostiffening [43], and wavelength selective protein release [44].

Despite these advances, the functional scope of light‐responsive hydrogels remains behind its theoretical potential and light control is predominantly limited to regulating crosslink density. Only in a few cases, light intensity is employed as a parameter to modulate crosslinking [1, 45, 46], and intermediate wavelengths of light are used to shift the photostationary state of reversible crosslinks [5]. However, viscoelastic properties are not solely determined by the thermodynamic equilibrium of the crosslinks but are critically governed by the dynamics of crosslink association and dissociation [47, 48, 49, 50, 51, 52]. Direct optical control over crosslink dynamics therefore represents an unexplored opportunity for photoregulated soft materials. Furthermore, most light responsive hydrogels rely on a single photoswitch, while multicolor control remains rare and is typically irreversible [8, 39, 53].

Here, we report a fully genetically encoded protein hydrogel that combines two visible light‐sensitive photoreceptors: Cph1, which forms reversible crosslinks under red light and dissociates under far‐red light, and CarH, which introduces green light cleavable crosslinks. Unexpectedly, Cph1‐based hydrogels exhibited an attenuated red light intensity response, forming stiffer hydrogels under low‐intensity red light than under higher intensities and continuing to stiffen after illumination ceases. This behavior arises from rapid, red light‐driven bidirectional photoisomerization that modulates crosslink lifetimes rather than crosslink density. To our knowledge, this is the first photoresponsive hydrogel in which the crosslink dynamics and not the photostationary state determine the material properties, giving rise to an attenuated light response with increasing intensity. Moreover, by combining Cph1 and CarH in a single material, we achieve reversible stiffness modulation using red and far‐red light alongside irreversible gel‐sol transitions induced by green light. This work introduces crosslink dynamics as a new axis of photoregulation and expands the design principles of multicolor, protein‐based light‐responsive hydrogels.

## 2 Results and Discussion

### 2.1 Hydrogel Design and Photoreceptor Integration

**FIGURE 1:**
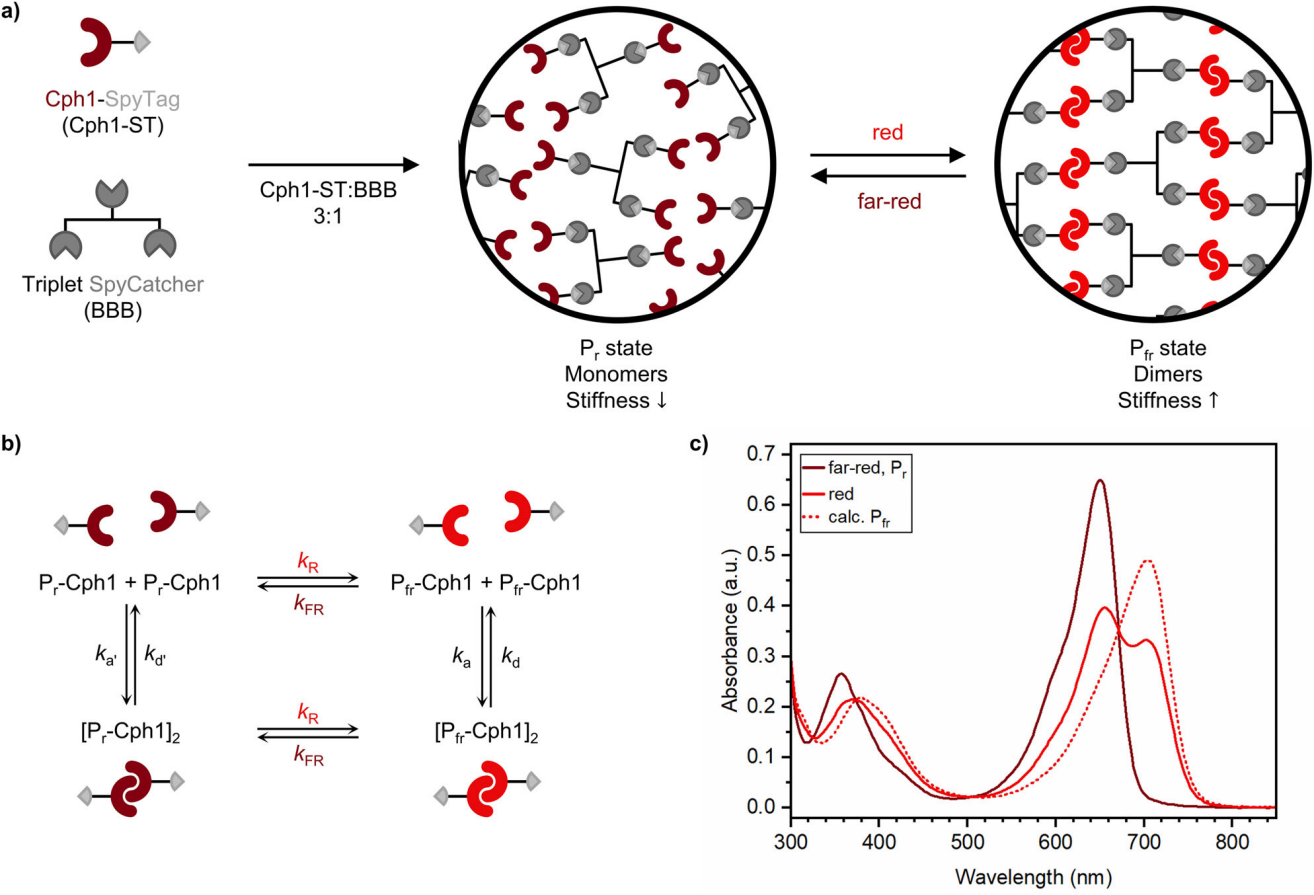
Dynamic photoswitching in Cph1‐based hydrogels. a) Upon mixing Cph1‐SpyTag (Cph1‐ST) with triplet SpyCatcher (BBB) in a molar ratio of 3:1, a covalently crosslinked backbone forms spontaneously. Illumination with red light induces Cph1 dimer assembly, leading to a non‐covalently crosslinked hydrogel and sol‐gel transition. Hydrogel formation is fully reversible by illumination with far‐red light, which triggers Cph1 dimer disassembly. b) Dynamics reaction network. Red light drives the P_r_→P_fr_ conversion with a rate constant *k*_R_, while far‐red light induces the P_fr_→P_r_ reversion with a rate constant *k*_FR_. While P_fr_‐Cph1 forms stable homodimers with an equilibrium constant K_R_ = *k*_a_/*k*_d_, far‐red light reverses this dimerization with an equilibrium constant K_FR_ = *k*_a’_/*k*_d’_. c) Absorbance spectrum of Cph1‐ST in the red light absorbing P_r_ state (far‐red solid line) with an absorbance maximum at 650 nm. Exposure to red light converts Cph1‐ST to the P_fr_ state and gives rise to a second peak at 700 nm (red solid line), resulting in a mixture P_r_ and P_fr_ states, n = 1. The calculated spectrum of the pure P_fr_ state is shown as a dashed red line.

In the hydrogel design, we opted for a fully protein‐based network assembled via genetically encoded click chemistry based on the reaction between SpyTag and SpyCatcher [30, 34]. This approach provides a straightforward and highly specific strategy for coupling multiple photoreceptors into a single hydrogel network. The triplet SpyCatcher protein (BBB), comprises three SpyCatcher domains connected by elastin‐like peptide (ELP) linkers, is not light‐responsive and serves as a modifiable backbone. As light‐responsive elements, the photoreceptors Cph1 and CarH were each fused to SpyTag peptides (Cph1‐ST and ST‐CarH, respectively; Figure 1a, shown for Cph1‐ST). Upon mixing SpyTag‐ and SpyCatcher‐containing proteins in a 3:1 molar ratio, spontaneous formation of covalent isopeptide bonds generates the hydrogel backbone, which can crosslink through light‐sensitive non‐covalent protein‐protein interactions.

As a red/far‐red light‐responsive element, we selected the N‐terminal domain of cyanobacterium phytochrome 1 (Cph1) from *Synechocystis* sp. PCC6803, fused to a C‐terminal SpyTag (Cph1‐ST). Cph1 includes a covalently attached tetrapyrrole chromophore (phycocyanobilin), which undergoes reversible photoisomerization between a red light absorbing P_r_ state (P_r_‐Cph1) and far‐red light absorbing P_fr_ state (P_fr_‐Cph1). Red light drives P_r_→P_fr_ conversion with a rate constant of *k*_R_, while far‐red light induces the reverse P_fr_→P_r_ reaction with a rate constant of *k*_FR_ (Figure 1b). Importantly, P_fr_‐Cph1 forms stable homodimers (equilibrium constant K_R_ = *k*_a_/*k*_d_), leading to red light‐induced crosslinks and sol‐gel transition. Illumination with far‐red light reverses dimerization (equilibrium constant K_FR_ = *k*_a’_/*k*_d_; K_R_ > K_FR_), resulting in gel‐sol transitions. In this study, we employed the R472A mutant of Cph1 to maximize the photodynamic range [27].

Purified Cph1‐ST under far‐red light exhibits a characteristic absorption maximum at 650 nm (P_r_ state), while upon red light illumination a second peak at 700 nm appears, corresponding to the P_fr_ state (Figure 1c). Under continuous red light illumination, Cph1 reaches a photostationary state (PSS) with a constant P_fr_:P_r_ ratio of approximately 32:68, independent of red light intensity (0.03‐35 mW/cm^2^) as determined from the absorbance spectra and the fluorescence of the P_r_ state (Figure S1). The substantial spectral overlap of the P_r_ and calculated P_fr_ states in the red light region leads to bidirectional P_r_⇄P_fr_ interconversion under red light, such that increasing light intensity accelerates both forward and backward reaction rates proportional to the light intensity but the ratio of the two rates, and hence the PSS composition remains constant (*k*_R_ = Φ_R_ · I_abs_; *k*_FR_ = Φ_FR_ · I_abs_; Φ is quantum yield and I_abs_ is the intensity of the absorbed light). Notably, when illumination is stopped the dynamics of the PSS is lost (at I_abs_ = 0, *k*_R_ = *k*_FR_ = 0) but the P_fr_:P_r_ ratio is maintained in the dark‐adapted state as long as thermal relaxation is negligibly slow. Illumination with far‐red light quantitatively converts P_fr_ back to P_r_. While the photoconversion P_fr_→P_r_ with far‐red light was rapid with a half‐life of 13 s, the thermal relaxation was extremely slow, with only about 3% of the protein reverting to the P_r_ state after 2.5 days in the dark (Figure S2).

### 2.2 Attenuated Light Intensity and Reversible Red/Far‐Red Light Response in Hydrogels

**FIGURE 2:**
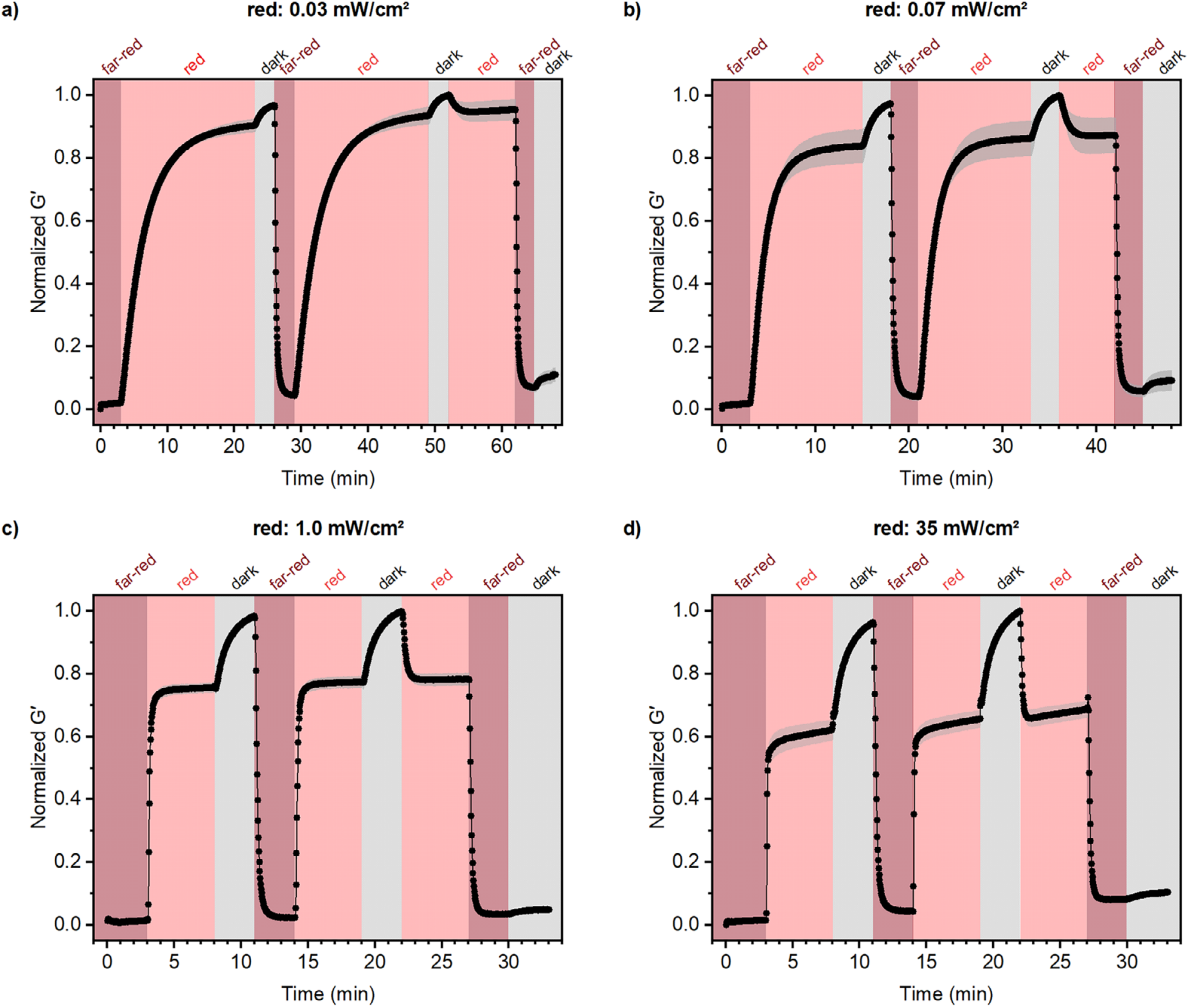
Photorheological characterization of Cph1‐based hydrogels. During the measurement, hydrogels were illuminated with far‐red light (shaded in dark red), red light (shaded in red), or kept in the dark (shaded in grey). While the far‐red light intensity was kept constant at 0.54 mW/cm^2^, different red light intensities of a) 0.03 mW/cm^2^, b) 0.07 mW/cm^2^, c) 1.0 mW/cm^2^, and d) 35 mW/cm^2^ were applied. Shown is the normalized mean of the storage modulus G' with corresponding standard deviation, n = 5, 4, 3, 3 for a, b, c, d, respectively.

We next investigated the mechanical properties of Cph1‐based hydrogels formed from a 3:1 mixture of Cph1‐ST and BBB using photorheological measurements in time‐sweep mode (Figures 2 and S3). Initially, under far‐red light, the material behaved as a viscous liquid with a low storage modulus (G'). Under red light illumination, G' increased, indicating hydrogel formation (Figure 2). For clarity, all data in Figure 2 were normalized to the maximum G' value obtained for the P_fr_‐Cph1 hydrogels in the dark, accounting for batch‐to‐batch variability in absolute stiffness (15‐90 Pa). We confirmed that on the same hydrogels identical final G' values were reached irrespective of the red light intensity used prior to dark adaptation and that the illumination with different light intensities alone does not alter G' in a control hydrogel without light‐responsive element (Figure S4).

Illumination with red light of different intensities (0.03‐35 mW/cm^2^) until G' reached a plateau revealed a striking dependence on light intensity (Figures 2a–d). Higher red light intensities led to a faster reaching of the plateau in G', consistent with accelerated P_r_→P_fr_ (*k*_R_) conversion and Cph1 dimerization. Unexpectedly, the lowest red light intensity produced the highest increase in G' at the plateau (Figure 2a), whereas high‐intensity illumination resulted in softer gels (Figure 2d). This attenuated response to red light contradicts the expectation that identical PSS should yield identical crosslink densities and mechanical properties and is the opposite of previous reports in which light intensity and response had a positive correlation until a maximum response was reached.

A second notable observation was that upon cessation of red light illumination, all Cph1‐hydrogels continued to stiffen in the dark, attaining a dark‐adapted state. The largest post‐illumination increase in G' occurred for gels previously exposed to the highest red light intensities, ultimately leading all gels to converge to the same final stiffness. Because thermal P_fr_→P_r_ conversion is negligible on this timescale, these changes cannot be attributed to altered P_fr_:P_r_ ratios. Illumination with far‐red light fully reversed this stiffening, restoring the sol state for P_r_‐Cph1.

Repeated red/far‐red light cycling reproduced identical G' values, showing full reversibility of hydrogelation and no sign of photodamage, also demonstrated in solution over 10 cycles (Figure S5). When these dark‐adapted P_fr_‐Cph1 hydrogels were illuminated with red light again, G' dropped to the same value observed during prior red light exposure, regardless of the initial state. The fact that the same G' values are obtained under red light illumination of a certain intensity no matter if one starts from P_fr_‐Cph1 or P_r_‐Cph1 states, indicates the existence of a dynamic PSS in which the hydrogel mechanics are governed by light controlled kinetic processes. We therefore hypothesized that the observed differences in G' under different intensities of red light and subsequently in the dark arise not from differences in crosslinking density (K_R_ and K_FR_ remain constant) but from changes in crosslink dynamics (*k*_a_, *k*_d_, *k*_a_, and *k*_d´_ in the equilibria), driven by light‐dependent modulation of forward and backward photoconversion rates rather than the equilibrium populations (*k*_R_ and *k*_FR_ increase proportional to light intensity but the ratio *k*_R_:*k*_FR_ remains constant). The coupled Cph1 dimerization equilibria seem sensitive to the increased dynamics of the PSS, which translates into changes in hydrogel properties.

### 2.3 Red Light Intensity Modulates Association and Dissociation Kinetics

**FIGURE 3:**
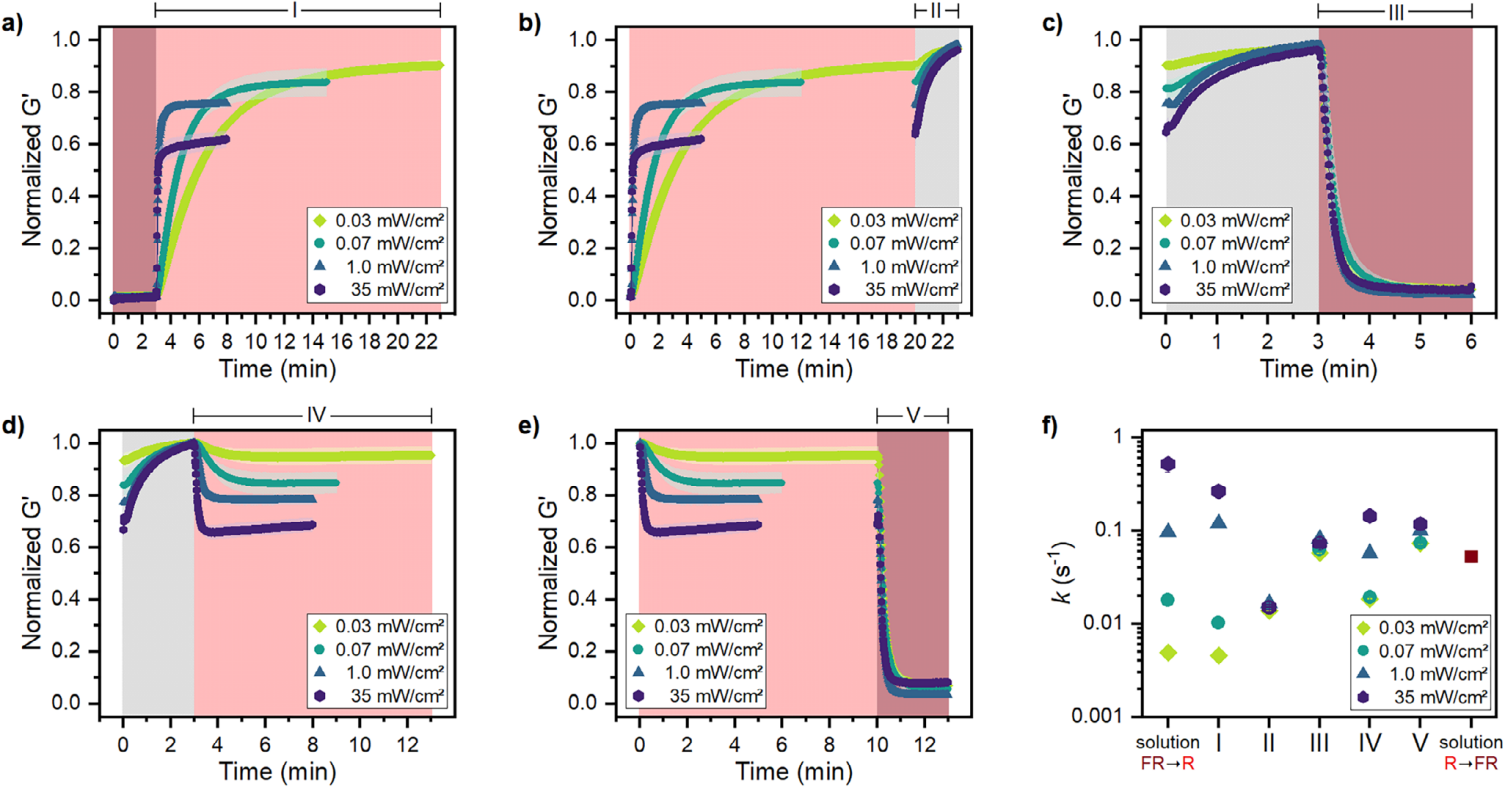
Photochemical response kinetics of Cph1 in solution and hydrogels. Data of Figure 2 was replotted to visualize the difference in G' for five distinct light transitions (a‐e, steps I‐V). The hydrogels were exposed to far‐red light (shaded in dark red), red light at different intensities (shaded in red), or kept in the dark (shaded in grey). f) Rate constants *k* were calculated for photoconversion in solution and in hydrogels for the different steps (I‐V).

To elucidate the molecular origin of the red light intensity‐dependent mechanical responses, we analyzed the reaction kinetics both at the molecular and hydrogel level. Time resolved vis spectra and fluorescence measurement for Cph1 in solution revealed that higher red light intensities accelerate the rate at which the PSS is approached without altering its composition, with apparent rate constants of 0.0049, 0.0178, 0.0962, and 0.5204 s^−1^ for 0.03, 0.07, 1.0, and 35 mW/cm^2^, respectively (Figure 3f solution FR→R, Figures S6, S8, Table S2). Correspondingly, rate constants for hydrogel stiffening obtained from the photorheology increased with red light intensity (0.0046, 0.0102, 0.1201, 0.2637 s^−1^) (Figure 3a,fI). Notably, the rate constants in solution and in the hydrogels were comparable, indicating that the photoswitching and not the formation of the Cph1 dimer‐based cross‐links is the rate‐limiting step (Figures 3f, S8, Table S2). Under high‐intensity red light the P_fr_→P_r_ photoreversion competes with P_fr_‐Chp1 association and the P_r_‐Cph1 dimers dissociate faster than they can be reconverted to the P_fr_ state. Therefore, increased bidirectional photoswitching also results in accelerated crosslink dynamics, as indicated by the lower final G' values obtained under higher red light illumination (Figure S7).

When red light illumination was stopped, photochemical interconversion is interrupted (*k*_R_ = *k*_FR_ = 0) and the competition between photoconversion and dimerization is removed, such that all hydrogels stiffen to the same extend, independent of prior illumination history and set by the P_fr_:P_r_ ratio at the PSS. The rate constants during this dark adaptation were identical for all samples (Figure 3b,fII), consistent with relation of dynamic crosslinks at the PSS to a less dynamic dark‐adapted equilibrium state.

Re‐illumination with red light restored the same dynamic state, again with a red light intensity‐dependent rate at which the dark‐adapted state converts to PSS (Figure 3d,fIV, 0.0186, 0.0194, 0.0576, 0.1441 s^−1^ for 0.03, 0.07, 1.0, and 35 mW/cm^2^, respectively). Likewise, far‐red light‐induced rapid softening processed with comparable kinetics (Figure 3fIII,V) regardless of whether gels originated from a dark‐adapted static (Figure 3c,fIII) or red light treated dynamic state (Figure 3e,fV). These rates closely matched the rate for photoreversion back to the P_r_ state in solution (Figure 3f solution R→FR, 0.0528 s^−1^), indicating that also during photosoftening photoconversion was the rate determining step and dimer dissociation proceeded faster.

**FIGURE 4:**
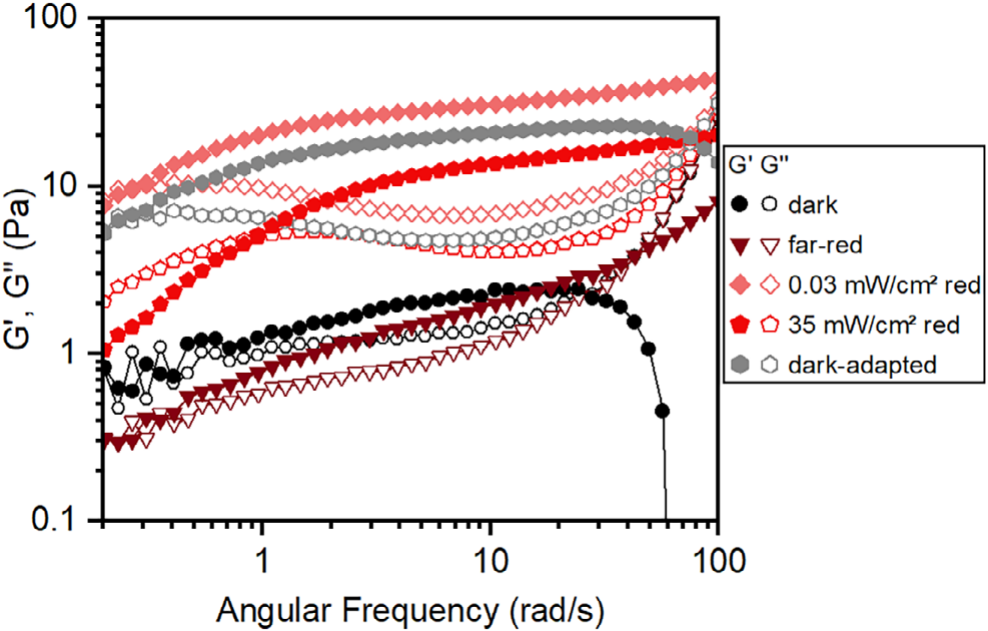
Frequency sweep measurements of Cph1 hydrogels in the dark (black circles), under far‐red light (0.54 mW/cm^2^, dark red triangles), low‐intensity red light (0.03 mW/cm^2^, pale red squares), high‐intensity red light (35 mW/cm^2^, red pentagons) and in the dark after illumination with high‐intensity red light (dark‐adapted state, grey hexagons). Full shapes represent G' and open shapes Gʺ, n = 1.

Additional evidence for light‐controlled crosslink dynamics was obtained from frequency sweep measurements in the linear viscoelastic region (at 3 rad/s), in which the low‐frequency crossover points of G' and Gʺ marks the transition from liquid‐like to solid‐like properties and is indicative of the crosslinking dynamics (Figure 4). Hydrogels under low‐intensity red light (0.03 mW/cm^2^) exhibited the highest plateau value of G' and the largest separation between storage (G') and loss modulus (Gʺ) with a low‐frequency crossover at only very low angular frequency (0.26 rad/s), characteristic of elastic solids. In contrast, high‐intensity red light (35 mW/cm^2^) resulted in lower G' and Gʺ values with an increase in the low‐frequency crossover point (0.90 rad/s), indicative of more dynamic crosslinks. When high‐intensity red light illumination was switched off, the low‐frequency crossover point shifted again to lower frequencies (0.25 rad/s) in the dark‐adapted state, confirming reduced crosslink dynamics. It should be noted that the storage moduli in the high frequency regime for these three hydrogels do not fully converge, suggesting that, in addition to their dynamics, the crosslinking density and backbone interactions may also be influenced by illumination to some extent. Control Cph1 samples kept in the dark or under far‐red light had similarly low moduli and early high‐frequency crossover points already at frequencies of 26.12 and 42.78 rad/s (dark and far‐red, respectively), showing a less crosslinked network overall.

Photorheological time‐sweep measurements in the viscous regime (i.e., at angular frequencies below the low‐frequency crossover point 0.1‐1.0 rad/s) confirm faster crosslink dynamics at higher red light intensities (Figure S9, S10). At these long‐time scales, the hydrogel network is in a relaxed state, and changes in G' and Gʺ upon red light exposure can be attributed solely to Cph1‐mediated crosslink formation. Even at very low angular frequencies (0.1 rad/s; Figure S10a), G' is lower at high red light intensity than at low red light intensity and shows dark‐adaptation once illumination is stopped. Notably, at 1 rad/s, the Chp1 hydrogel exhibits solid‐like behavior (G' > Gʺ) at low red light intensity and liquid‐like behavior (G' < Gʺ) at high red light intensity (Figure S10d), further supporting the conclusion that Cph1‐based crosslinks become more dynamic with increasing red light intensity.

### 2.4 Combining Reversible Red/Far‐Red Light and Irreversible Green Light Response

**FIGURE 5:**
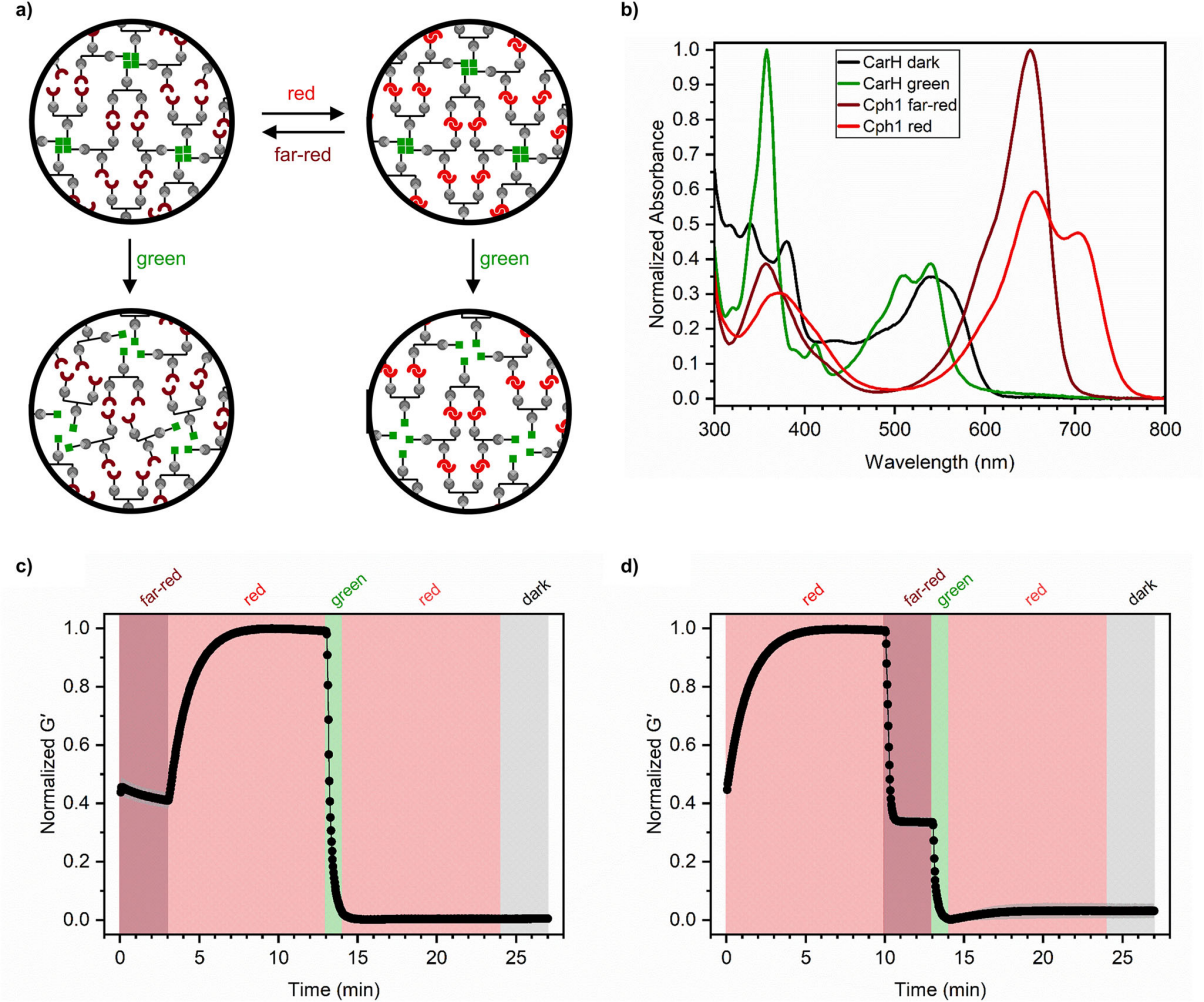
Reversible and irreversible multicolor light response in combined Cph1‐CarH hydrogels. a) Crosslinking structure in Cph1‐CarH hydrogels. Red and far‐red light addresses reversible Cph1 crosslinks, whereas green light triggers an irreversible gel‐sol transition due to CarH tetramer disassembly. b) Absorbance spectra of CarH in the dark (black solid line) and after green light exposure (green solid line) overlayed with absorbance spectra of Cph1 under far‐red (dark red solid line) and red (red solid line) light, n = 1. c‐d) Photorheological measurements demonstrate the increase and decrease in stiffness with red (shaded in red, 0.07 mW/cm^2^) and far‐red (shaded in dark red, 0.54 mW/cm^2^) light illumination, respectively, and rapid irreversible gel‐sol transition after green light illumination (shaded in green, 0.8 mW/cm^2^) independent of prior c) red or d) far‐red light. Data is shown as mean with corresponding standard deviation, n = 3.

An important goal in light‐responsive hydrogel design is independent control of multiple material properties using orthogonal wavelengths. The modular SpyTag‐SpyCatcher click‐chemistry of the hydrogel backbone enables straightforward integration of additional photoresponsive elements. To demonstrate this, we incorporated the C‐terminal adenosyl cobalamin‐binding domain of CarH from *Thermus thermophilus* with a N‐terminal SpyTag (ST‐CarH) as a green light‐responsive element (Figure 5b). In CarH‐only‐hydrogels formed from BBB and ST‐CarH, addition of adenosyl cobalamin (AdoCbl) induces CarH tetramerization and gel formation in the dark, while green light irreversibly dissociates tetramers, leading to rapid gel‐sol transitions [30, 34].

We combined ST‐CarH, Cph1‐ST, and BBB in a 1:2:1 molar ratio (i.e., equal molar SpyTag‐SpyCatcher groups) (Figure 5a). Upon AdoCbl addition, hydrogels even formed under far‐red light illumination due to CarH crosslinking and stiffened further under red light (0.07 mW/cm^2^) through additional Cph1‐meditated crosslinks (Figure 5c). Likewise, when these gels were first exposed to red light, they were stiffer and softened with far‐red light illumination (Figure 5d). In both cases, green light induced rapid and irreversible gel‐sol transitions, and their initial stiffness could not be restored with red light, showing that Cph1 crosslinks alone are insufficient to maintain network integrity. For comparison, pure Cph1 hydrogels exposed to red light did not undergo green light induced softening and only a slight increase in G′ was observed similar to the dark‐adapted state (Figure S11). Interestingly, Cph1 hydrogels switched from far‐red to green light illumination stiffened, which we attributed to residual P_r_ absorption and spectral overlap of the light sources of the P_r_ in the green and the red light contamination of the green light source used here (Figure S12). On the other hand, pure CarH hydrogels responded exclusively to green light and neither red nor far‐red light illumination had an effect on their mechanical properties and absorbance spectra (Figure S13). Together, the reversible red/far‐red response of Cph1 and irreversible green light response of CarH enables multicolor, multimodal control over hydrogel mechanics, significantly expanding the functional design space of light responsive biomaterials.

## 3 Conclusion

In conclusion, we have developed a fully protein‐based hydrogel system that responds to red, far‐red and green light through wavelength‐specific and intensity‐dependent changes in mechanical properties. Reversible Cph1‐mediated crosslinks enable reversible dynamic modulation of hydrogel stiffness under red and far‐red light, while CarH‐based crosslinks induce irreversible green light‐triggered gel‐sol transitions. The highly modular SpyTag‐SpyCatcher chemistry used to assemble the hydrogel backbone provides a versatile platform for introducing diverse photoswitchable proteins, as demonstrated here for Cph1 and CarH, and could readily be extended to additional photoreceptors responsive to blue or UV light [29, 54, 55].

A key finding of this work is the attenuated red light intensity response of Chp1 based hydrogels and subsequent dark‐adaptation, in which higher light intensities produced softer hydrogels due to accelerated dynamics in the PSS and increased crosslinking dynamics. Although this intensity‐dependent attenuation in the Cph1 hydrogels appears to contradict previous optogenetic studies with phytochrome photoreceptors reporting monotonic responses to red light intensity [56, 57, 58], it aligns with the observations of the red light‐induced protein‐protein interactions between plant phytochrome B of *Arabidopsis thaliana* (*At*PhyB) and phytochrome‐interacting factors (PIFs) [59]. Möglich et al. demonstrated that continuous red light illumination (1‐69 mW/cm^2^) decreases PhyB‐PIF complexation at higher intensities due to accelerated P_r_⇄P_fr_ interconversion and complex dissociation rates. In fact, the intensity‐dependent binding dynamics have also been observed in the context of PhyB‐PIF mediated T‐cell receptor activation [60]. Our results extend this attenuation mechanism from the molecular interactions and the optogenetic regulation of gene expression and T‐cell activation to macroscopic material properties.

This behavior demonstrates that hydrogel mechanics can be governed by light‐controlled crosslink lifetimes, thereby introducing a kinetic dimension to photoregulated soft materials. Similar modulation of PSS dynamics may be achieved in other photoswitchable hydrogels [1, 15, 16], provided that bidirectional photoisomerization rather than crosslink formation is the rate limiting step. Beyond expanding the toolbox of light‐responsive hydrogels, these results establish PSS dynamics as a general design principle for multicolor‐responsive, protein‐based materials. In particular, such materials enable dynamic and reversible control over viscoelastic cues in 3D cell culture systems, offering new opportunities to probe mechanotransduction [16, 31, 32, 61, 62, 63], cell differentiation, and tissue morphogenesis with high spatiotemporal precision.

## Author Contributions

**Saskia Frank**: conceptualization, methodology, investigation, data curation, formal analysis, visualization, writing – original draft. **Seraphine V. Wegner**: conceptualization, investigation, supervision, funding acquisition, project administration, writing – original draft.

## Conflicts of Interest

The authors declare no conflicts of interest.

## Supporting information




**Supporting Information**: The authors have cited additional references within the Supporting Information [64, 65, 66].

## Data Availability

The data that support the findings of this study are available from the corresponding author upon reasonable request.
