# Supplementary material for: Multicolor and Attenuated Light Intensity Responses in Protein Hydrogels Arising from Photoregulated Crosslink Dynamics
Source: Angew Chem Int Ed Engl. 2026 May 28;65(31):e6692301. doi: 10.1002/anie.6692301 (PMC13411494; doi:10.1002/anie.6692301)
Supplement: Supplementary file 1 — Supporting Information: The authors have cited additional references within the Supporting Information [64, 65, 66]. [file ANIE-65-e6692301-s001.pdf]

Supporting Information

Multicolour and Attenuated Light Intensity Responses in Protein Hydrogels Arising from Photoregulated Crosslink Dynamics

Saskia Frank[a] and Seraphine V. Wegner*[a]

[a] S. Frank, Prof. S. V. Wegner
Institute of Physiological Chemistry and Pathobiochemistry
University of Münster
Waldeyerstraße 15, 48149 Münster, Germany
E-mail: wegnerse@uni-muenster.de

Abstract: Light-responsive hydrogels enable non-invasive, precise, remote control over material properties with great biocompatibility, yet achieving multicolour addressability and dynamic regulation of mechanical properties remains challenging. Most systems rely on single photoswitches and regulate stiffness primarily through changes in crosslinking density. Here, we present a fully protein-based hydrogel that combines multicolour light responsiveness with optical control of crosslink dynamics. The hydrogel includes two visible light responsive photoceptors: the cyanobacterial phytochrome Cph1, which enables reversible red/far-red light-controlled crosslinking, and CarH, which introduces irreversible green light induced gel-sol transitions. Remarkably, Cph1-based hydrogels exhibited an attenuated red light intensity response, forming stiffer networks under low intensity illumination than under high intensity light and show a subsequent dark-adaptation with stiffening once red light illumination is stopped. This counterintuitive behaviour arises from light-driven bidirectional photoisomerization that modulates crosslink lifetimes without altering the photostationary state composition. Together, these findings establish orthogonally addressable reversible and irreversible crosslinks and photoregulated crosslink dynamics as new design principles for multicolour light responsive biomaterials.

Table of Contents

Experimental Section S2
References S4
Supporting Figures and Tables S5

Experimental Section

Materials: Phycocyanobilin (PCB, SC-1800) was purchased from Sirius Fine Chemicals (SiChem GmbH, Bremen, Germany) and adenosyl cobalamin (Coenzyme B_{12}, C0884) from Sigma-Aldrich® (Merck KGaA, Darmstadt, Germany). All other chemicals were purchased from Sigma-Aldrich® (Merck KGaA, Darmstadt, Germany) or Carl Roth (Carl Roth GmbH + Co. KG, Karlsruhe, Germany), if not stated otherwise.
Buffers were prepared with double distilled water, filtered (0.2 µm cellulose nitrate; Carl Roth. Karlsruhe, Germany) and degassed prior to use.

Emission spectra and intensity determination of external light sources: Peak emission values for each light source were determined by UV/vis spectroscopy with a spectrophotometer (DeNovix DS-11 FX+, DeNovix Inc., Wilmington, USA). A 10 mm PS cuvette was filled with double distilled water and the lamp positioned such that the light beam pathed through the water. The lowest value of the data represents the peak emission value of the respective light source. The negative data was converted to positive values and normalised. Data for the green lamp was smoothed using the method moving average in the Origin software (OriginPro 2022 SR1, 9.9.0.225). The maximal emission wavelength for green, red and far-red light were determined to be 520 nm, 625 nm and 730 nm, respectively.
Prior to each experiment, the light intensity was measured with an USB Power Meter PM16-120 (Thorlabs GmbH, Bergkirchen, Germany) and the corresponding Optical Parameter Monitor software (PM 230201114).

Protein Expression and Purification: The construct encoding the gene for Cph1 R472A with a C-terminal added elastin-like polypeptide (ELP), SpyTag and poly-histidine-tag (His_6-tag) (Cph1-ST, 68.2 kDa), was synthesized and cloned into a pCDFDuet-1 vector between the NcoI and NotI in the MCS1 (multiple cloning site 1) by GenScript (GenScript Biotech (Netherlands) B.V., Rijswijk, Netherlands). The pQE80l plasmids harbouring the genes for His_6-tagged SpyTag-ELP-$CarH_C$ (ST-CarH, 32.2 kDa), His_6-tagged triplet SpyCatcher (BBB, 54.9 kDa) and His_6-tagged triplet Spy Tag (AAA, 20.8 kDa) were a gift of Fei Sun (Hong Kong, China).[1–3] The respective amino acid sequences are listed in Table S1.[4] Plasmids harbouring the respective genes were transformed into *E. coli* BL21(DE3) cells by heat shock and transformed clones were selected by 100 µg/mL ampicillin and 50 µg/mL streptomycin, for pQE80l and pCDFDuet-1 vectors, respectively, on lysogeny broth (LB) agar plates. Bacteria cultures, inoculated with 15 mL pre-culture grown from a single colony, were grown at 37 °C, 250 rpm in 1 L LB medium, supplemented with 100 µg/mL ampicillin and 50 µg/mL streptomycin, respectively, until they reached an optical density at 600 nm of 0.6-0.8. After induction with 500 µM isopropyl β-*D*-1-thiogalactopyranoside (IPTG), proteins were expressed at 16 °C overnight. The next day, cells were harvested by centrifugation (20 min, 6000 rcf, 4 °C; Avanti™ J-25 with rotor JA-10, Beckman Coulter Life Sciences, Brea, USA) and bacteria pellets frozen at -20 °C prior to protein purification. For purification, bacteria pellets from 2 L expression culture were resuspended in 80 mL working buffer (50 mM Tris-HCl, pH 7.4, 300 mM NaCl) containing 20 mM imidazole and 1 mM phenylmethylsulfonyl fluoride (PMSF), lysed by ultrasound (3x 5 min, 50 % amplitude; Omni Sonic Ruptor 400 (Omni International, Inc., Kennesaw, USA) supplied with a 12.7 mm processing tip 21-OR-T-500 (biolabproducts GmbH, Bebensee, Germany)) and the supernatant cleared by centrifugation (1 h, 17500 rcf, 4 °C; Avanti™ J-25 with rotor JA-25.50). In case of Cph1-ST, PCB was added to a final concentration of 100 µM to the supernatant and incubated for 1 h on ice before continuing with the purification. All protein solutions were filtered (0.45 µm PES membrane) and loaded onto a HisPrep FF 16/10 column (Cytiva Europe GmbH, Freiburg, Germany) and purified using a NGC chromatography system (Bio-Rad Laboratories GmbH, Feldkirchen, Germany). After washing with 5 column volumes (CV) working buffer with 20 mM imidazole and 1 CV with 40 mM imidazole, the protein was eluted with an imidazole gradient ranging from 40-300 mM imidazole over 5 CV, with a final step over 1 CV with 500 mM imidazole. Protein purity was analysed by SDS-PAGE (Figure S14) and pure protein fractions were combined and dialysed extensively into distilled water at 4 °C. Possible insoluble protein aggregates after dialysis were cleared out by centrifugation (40 min, 4500 rcf, 4 °C; Centrifuge 5804 R, Eppendorf SE, Hamburg, Germany), the supernatant then flash frozen in liquid N_2 and freeze-dried at -80 °C for 2 days. Proteins were stored at -20 °C for short-term storage before further use.

Hydrogel Synthesis: Lyophilized proteins were dissolved in working buffer (50 mM Tris-HCl, pH 7.4, 300 mM NaCl) overnight at 4 °C to aim a final concentration of 10 % (w/v). Undissolved protein aggregates were removed by centrifugation (10 min, 16900 rcf, 4 °C; Centrifuge 5418 R, Eppendorf SE, Hamburg, Germany) and protein

concentration was determined by spectrophotometry (DeNovix DS-11 FX+, DeNovix Inc., Wilmington, USA). To form a pure red/ far-red light sensitive hydrogel, BBB was added to a low bind Eppendorf tube and then mixed with Cph1-ST at a molar ratio of 3:1 at room temperature (final concentration BBB 0.38 mM, Cph1-ST 1.15 mM). For red/far-red and green light sensitive hydrogels, BBB was mixed with Cph1-ST at a molar ratio of 1:2 and 1 eq. ST-CarH was added (final molar ratio 2:1:1 Cph1-ST:BBB:ST-CarH). Green light sensitive hydrogels were formed by mixing BBB with ST-CarH in a molar ratio of 1:3. Gelation through tetramerization of CarH monomers was initiated by addition of adenosyl cobalamin (AdoCbl, 5 mM in distilled water; molar ratio 1:1 AdoCbl:ST-CarH) in the dark (final concentrations of hydrogels after addition of AdoCbl for red/far-red/green light sensitive hydrogels: BBB 0.39 mM, Cph1-ST 0.79 mM, ST-CarH 0.39 mM; for green light sensitive hydrogels: BBB 0.40 mM, ST-CarH 1.19 mM). Light unresponsive hydrogels were formed through mixing of BBB and AAA in a 1:1 molar ratio (final concentrations BBB 0.98 mM, AAA 0.98 mM). All gels were cured at room temperature overnight in the dark.

Rheology: Rheological measurements were performed on a Modular Compact Rheometer MCR 102 (Anton Paar GmbH, Graz, Austria) with cone and plate configuration, equipped with a P-PTD200 + H-PTD200 photo cell (Anton Paar, Graz, Austria) and Anton Paar RheoCompass V1.20.40.496 analysis software (Anton Paar GmbH, Graz, Austria). Typically, hydrogels of 30-40 µL were analysed. For time sweep measurements, hydrogels were placed on the lower glass plate of the rheometer and compressed with the upper steel cone (CP20-1, 20 mm diameter, Anton Paar) to the final measurement position under far-red light (730 nm, 0.54 mW/cm²). After 2 min illumination, samples were continuously measured at 20 °C, with 5 % shear strain and 1 and 3 rad/s angular frequency (2 rad/s angular frequency for AAA-BBB hydrogels), respectively. Illumination patterns were applied during the measurement with different intensities of red light (625 nm, 0.03, 0.07, 1.0, 35 mW/cm²), green light (520 nm, 0.8 mW/cm²) and far-red light (730 nm, 0.54 mW/cm²), respectively, or kept in the dark. The mean of at least 3 independent gels was calculated and min-max normalized using equation 1, then plotted and shown with the corresponding standard deviation, where x_{min} was the initial value under far-red light and x_{max} the maximal value in the dark-adapted state.

$$x_{normalized} = \frac{x - x_{min}}{x_{max} - x_{min}} \qquad (1)$$

Additionally, single measurements of Cph1-ST-BBB hydrogels were performed at 0.1, 0.3 and 0.5 rad/s.

For Frequency sweep measurements, hydrogels were either kept in the dark or pre-incubated in the final measurement position under red light of different intensities (0.03 mW/cm² for 20 min, 35 mW/cm² for 5 min), under far-red light (0.54 mW/cm² for 5 min), under green light (0.8 mW/cm² for 8 min) or under high intensity red light (35 mW/cm² for 5 min) followed by 3 min in the dark. Samples were continuously measured at 20 °C, with a constant shear strain of 5 % and an angular frequency ranging from 0.1 to 100 rad/s with logarithmic spacing. During the measurement, gels were continuously illuminated from underneath the glass plate with the respective intensity of red, far-red and green light or kept in the dark. Data is shown for a single measurement per condition.

Determination of Light-dependent Kinetics: In order to determine the velocity at which the stiffness of the hydrogel increases or decreases and thus obtain information about the association and dissociation kinetics of Cph1-ST, rheological data at the specific light transitions was fitted to a single-exponential function using the Fit-o-mat software (GNU General Public License v3.0, version 0.973)[5]

$$F(t) = F_0 + F_1 \cdot e^{(-k \cdot t)} \qquad (2)$$

where F_0 and F_1 are normalized ΔG' amplitudes and *k* represents the rate constant for each process at the specific light transitions.

Absorbance Measurements: Absorbance spectra of 100 µM Cph1-ST in working buffer were recorded at 22 °C on a Tecan Spark® multimode microplate reader (Tecan Austria GmbH, Grödig, Austria) in triplicates. To follow the $P_r \rightarrow P_{fr}$ conversion, Cph1-ST was saturated with far-red light (730 nm, 0.54 mW/cm²) and absorbance spectra were recorded after illumination with red light (625 nm) at intensities of 0.03, 0.07, 1.0 and 35 mW/cm² for 1 s, 3 s, 5 s, 10 s, 20 s, 30 s, 1 min, 3 min, 5 min, 10 min and 20 min. $P_{fr} \rightarrow P_r$ photoreversion was recorded

analogously after the sample was initially saturated with red light (625 nm, 0.07 mW/cm²) and stepwise exposed to far-red light (730 nm, 0.54 mW/cm²). Data is shown as the mean of triplicates with corresponding standard deviation. The absorbance over time was fitted to Equation 2.
The dark recovery of Cph1-ST was followed for 2.5 days. For this, an absorbance spectrum of 100 µM Cph1-ST was recorded after saturating illumination with red light (625 nm, 0.07 mW/cm²). Absorbance at 650 nm and 700 nm was followed in the dark with a final absorbance spectrum recorded after 2.5 days.
Additional absorbance spectra were recorded of Cph1-ST after pre-illumination with far-red light (730 nm, 0.54 mW/cm²) followed by saturating exposure to green light (520 nm, 0.8 mW/cm²). Data is shown as the mean of triplicates with corresponding standard deviation.
$P_r \rightleftarrows P_{fr}$ photoswitching efficiency was tested at 22 °C with repeated cycles (n=10) of red and far-red illumination. The absorbance at 650 nm was measured first after saturating illumination with far-red light (0.54 mW/cm²) and second after saturating illumination with red light (1.0 mW/cm²). Data is shown as mean of three measurements with corresponding standard deviation.

With the above-mentioned settings, absorbance spectra of 100 µM ST-CarH (1:1 AdoCbl) were recorded in the dark, after 3 min illumination with far-red (0.54 mW/cm²), 10 min with red (0.07 mW/cm²) and after 1 min illumination with green light (0.08 mW/cm²). Data is shown as the mean of triplicates with corresponding standard deviation.

Fluorescence Measurements: Fluorescence intensity scans (λ_{ex}: 650 nm, λ_{em}: 665-700 nm) of 100 µM Cph1-ST in working buffer were recorded at 22 °C in a Tecan Spark® multimode microplate reader (Tecan Austria GmbH, Grödig, Austria) in triplicates. The P_r spectrum was recorded after saturating illumination with far-red light (730 nm, 0.54 mW/cm²), the photo stationary state (PSS) spectrum after saturating illumination with red light (625 nm, 0.07 mW/cm²). The ratio of the fluorescence of the P_r and PSS at the maximal emission peak at 677 nm was used to estimate the P_r : P_{fr} ratio in the PSS assuming that only the P_r state has an emission and was determined to be 32:68. Furthermore, the ratio of P_r:P_{fr} in the PSS was plotted versus the emission wavelength and fitted with a linear fit. The value for 677 nm was used to calculate the P_r contribution in the recorded P_{fr} spectrum and to correct the P_{fr} spectrum to obtain the spectrum of Cph1-ST with 100 % switching efficiency.
The P_r state fluorescence at excitation wavelength of 650 ± 5 nm and emission wavelength of 677 ± 5 nm of 100 µM Cph1-ST in working buffer at 22 °C was monitored over time. To follow the $P_r \rightarrow P_{fr}$ conversion, the sample was first saturated with far-red light (730 nm, 0.54 mW/cm²), then exposed to red light (625 nm) at intensities of 0.03, 0.07, 1.0 and 35 mW/cm² for 1 s, 3 s, 5 s, 10 s, 20 s, 30 s, 1 min, 3 min, 5 min, 10 min and 20 min. To monitor the $P_{fr} \rightarrow P_r$ reversion, Cph1-ST was pre-illuminated with red light (625 nm, 0.07 mW/cm²) and exposed to far-red light (730 nm, 0.54 mW/cm²) for 1 s, 3 s, 5 s, 10 s, 20 s, 30 s, 1 min, 3 min, 5 min, 10 min and 20 min. Data is shown as the mean of triplicates with corresponding standard deviation. The fluorescence over time was fitted to Equation 2.

References

[1] Z. Yang, Y. Yang, M. Wang, T. Wang, H. K. F. Fok, B. Jiang, W. Xiao, S. Kou, Y. Guo, Y. Yan, X. Deng, W.-B. Zhang, F. Sun, "Dynamically Tunable, Macroscopic Molecular Networks Enabled by Cellular Synthesis of 4-Arm Star-like Proteins", *Matter* **2020**, *2*, 233.

[2] F. Sun, W.-B. Zhang, A. Mahdavi, F. H. Arnold, D. A. Tirrell, "Synthesis of bioactive protein hydrogels by genetically encoded SpyTag-SpyCatcher chemistry", *Proc. Natl. Acad. Sci. U. S. A.* **2014**, *111*, 11269.

[3] S. Kou, Z. Yang, J. Luo, F. Sun, "Entirely recombinant protein-based hydrogels for selective heavy metal sequestration", *Polym. Chem.* **2017**, *8*, 6158.

[4] S. Frank, S. V. Wegner, "Programming Green Light-Responsive Hydrogels: From Photo-Weakening to Photo-Unresponsiveness and Photo-Strengthening", *Chem. Eur. J.* **2025**, *31*, e202404360.

[5] A. Möglich, "An Open-Source, Cross-Platform Resource for Nonlinear Least-Squares Curve Fitting", *J. Chem. Educ.* **2018**, *95*, 2273.

Supporting Figures and Tables

Table S1. Amino acid sequence and corresponding molecular weight (in Da) and molar extinction coefficient (in $M^{-1}cm^{-1}$) of proteins used in this study. The sequence of His_6-tag is underlined, the RGD binding motif written in bold. Sequences for SpyTag, SpyCatcher, elastin-like polypeptide (ELP), $CarH_C$ and Cph1 R472A are highlighted in yellow, cyan, light grey, green and red, respectively.

Protein Name	Molecular Weight (Da)	ε_{280} (M^{-1} cm^{-1})	Amino Acid Sequence (N-terminus → C-terminus)
BBB	54900.97	53860	MKGSSHHHHHHVDIPTTENLYFQGAMVDTLSGLSSEQG<br>QSGDMTIEEDSATHIKFSKRDEDGKELAGATMELRDSSG<br>KTISTWISDGQVKDFYLYPGKYTFVETAAPDGYEVATAIT<br>FTVNEQGQVTVNGKATKGDAHIDGPQGIWGQLDGHGVG<br>VPGVGVPGVGVPGEGVPGVGVPGVGVPGVGVPGVGVP<br>GEGVPGVGVPGVGVPGVGVPGVGVPGEGVPGVGVPGV<br>GELGRGDDSATHIKFSKRDEDGKELAGATMELRDSSGK<br>TISTWISDGQVKDFYLYPGKYTFVETAAPDGYEVATAITF<br>TVNEQGQVTVNGTSVPGVGVPGVGVPGEGVPGVGVPG<br>VGVPGVGVPGVGVPGEGVPGVGVPGVGVPGVGVPGVG<br>VPGEGVPGVGVPGVGVPGGLLDIPTTENLYFQGAMVDT<br>LSGLSSEQGQSGDMTIEEDSATHIKFSKRDEDGKELAGA<br>TMELRDSSGKTISTWISDGQVKDFYLYPGKYTFVETAAP<br>DGYEVATAITFTVNEQGQVTVNGKATKGDAHIDGPQGIW<br>GQLEWKK*
Cph1-SpyTag	68166.68	59735	MATTVQLSDQSLRQLETLAIHTAHLIQPHGLVVVLQEPDL<br>TISQISANCTGILGRSPEDLLGRTLGEVFDSFQIDPIQSRL<br>TAGQISSLNPSKLWARVMGDDFVIFDGVFHRNSDGLLVC<br>ELEPAYTSDNLPFLGFYHMANAALNRLRQQANLRDFYDV<br>IVEEVRRMTGFDRVMLYRFDENNHGDVIAEDKRDDMEP<br>YLGLHYPESDIPQPARRLFIHNPIRVIPDVYGVAVPLTPAV<br>NPSTNRAVDLTESILRSAYHCHLTYLKNMGVGASLTISLIK<br>DGHLWGLIACHHQTPKVIPFELRKACEFFGRVVFSNISAQ<br>EDTETFDYRVQLAEHEAVLLDKMTTAADFVEGLTNHPDR<br>LLGLTGSQGAAICFGEKLILVGETPDEKAVQYLLQWLENR<br>EVQDVFFTSSLSQIYPDAVNFKSVASGLLAIPIARHNFLLW<br>FRPEVLQTVNWGGDPNHAYEATQEDGKIELHPAQSFDL<br>WKEIVRLQSLPWQSVEIQSALALKKAIVNLILRQAEEGLVP<br>GVGVPGVGVPGEGVPGVGVPGVGVPGVGVPGVGVPGE<br>GVPGVGVPGVGVPGVGVPGVGVPGEGVPGVGVPGVGV<br>PGELG**RGD**TSAHIVMVDAYKPTKVDGLLEHHHHHH*
SpyTag-CarH	32182.16	22460	MKGSSHHHHHHVDAHIVMVDAYKPTKLDGHGVGVPGV<br>GVPGVGVPGEGVPGVGVPGVGVPGVGVPGVGVPGEGV<br>PGVGVPGVGVPGVGVPGVGVPGEGVPGVGVPGVGELP

			EDLGTGLLEALLRGDLAGAEALFRRGLRFWGPEGVLEHLLLPVLREVGEAWHRGEIGVAEEHLASTFLRARLQELLDLAGFPPGPPVLVTTPPGERHEIGAMLAAYHLRRKGVPALYLGPDTPLPDLRALARRLGAGAVVLSAVLSEPLRALPDGALKDLAPRVFLGGQGAGPEEARRLGAEYMEDLKGLAEALWLPRGPEKEAI*
AAA	20777.11	9970	MKGSSHHHHHHVDAHIVMVDAYKPTKLDGHGVGVPGVGVPGVGVPGEGVPGVGVPGVGVPGVGVPGVGVPGEGVPGVGVPGVGVPGVGVPGVGVPGEGVPGVGVPGVGELAHIVMVDAYKPTKTSVPGVGVPGVGVPGEGVPGVGVPGVGVPGVGVPGVGVPGEGVPGVGVPGVGVPGVGVPGVGVPGEGVPGVGVPGVGVPGGLLDAHIVMVDAYKPTKLEWKK*

Table S2. Rate constants in solution and hydrogels. Absorbance (at 650 and 700 nm), fluorescence and rheological data was fitted to a single exponential function (equation 2) to determine the rate constant *k* (n = 3 ± S.D.). Data is shown graphically in Figure S6 and S8.

Illumination	Light intensity	k (s^{-1})			
		A650	A700	Fluorescence	Rheology
far-red	0.54 mW/cm²	0.0528 ± 0.0011	0.0491 ± 0.0019	0.0540 ± 0.0033	0.0733 ± 0.0009[a]
red	0.03 mW/cm²	0.0049 ± 0.0002	0.0058 ± 0.0004	0.0046 ± 0.0003	0.0046 ± 0.00001[b]
red	0.07 mW/cm²	0.0178 ± 0.0013	0.0180 ± 0.0006	0.0141 ± 0.0012	0.0102 ± 0.00003[b]
red	1.0 mW/cm²	0.0962 ± 0.0027	0.0800 ± 0.0081	0.1065 ± 0.0062	0.1201 ± 0.0012[b]
red	35 mW/cm²	0.5204 ± 0.0929	0.7541 ± 0.1849	0.7193 ± 0.2453	0.2637 ± 0.0059[b]

[a] Value corresponds to data in Figure 3f V 0.03 mW/cm².
[b] Values correspond to data in Figure 3f I.

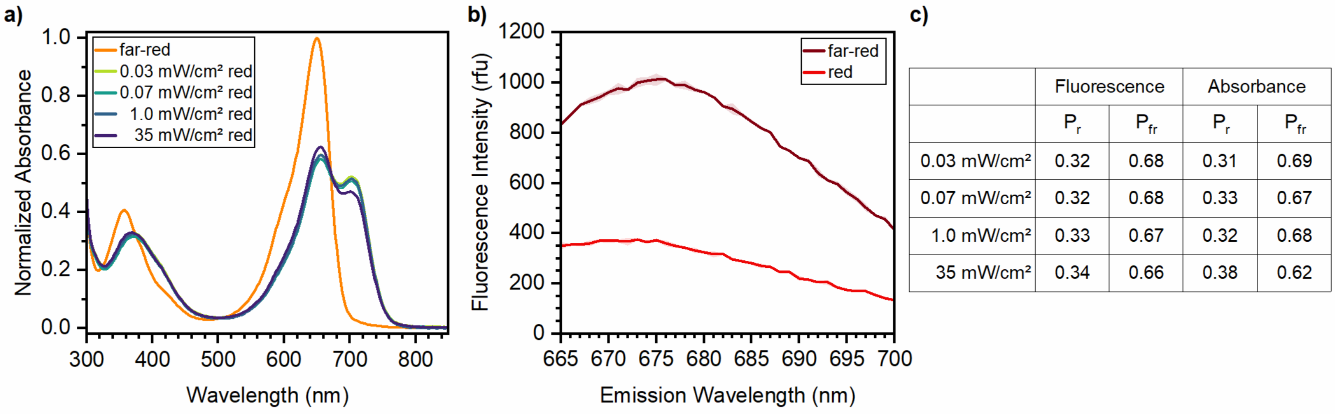

c)

	Fluorescence		Absorbance	
	P_r	P_{fr}	P_r	P_{fr}
0.03 mW/cm²	0.32	0.68	0.31	0.69
0.07 mW/cm²	0.32	0.68	0.33	0.67
1.0 mW/cm²	0.33	0.67	0.32	0.68
35 mW/cm²	0.34	0.66	0.38	0.62

Figure S1. Spectroscopic analysis of Cph1-ST after red and far-red light illumination. a) Absorbance spectra of Cph1-ST at 22 °C at the PSS. After pre-illumination with far-red light (0.54 mW/cm², orange line), Cph1-ST was saturated with red light exposure at intensities of 0.03 mW/cm² (light green line), 0.07 mW/cm² (blue-green line), 1.0 mW/cm² (dark blue line) and 35 mW/cm² (purple line). b) Fluorescence spectra of Cph1-ST at 22 °C after far-red light illumination (P_r state, dark red line) and after saturating red light illumination until PSS (mixture of P_r:P_{fr}, red line). Data in a) and b) is shown as mean values with standard deviation, n = 3. c) Distribution of Cph1-St in P_r and P_{fr} according to fluorescence and absorbance data.

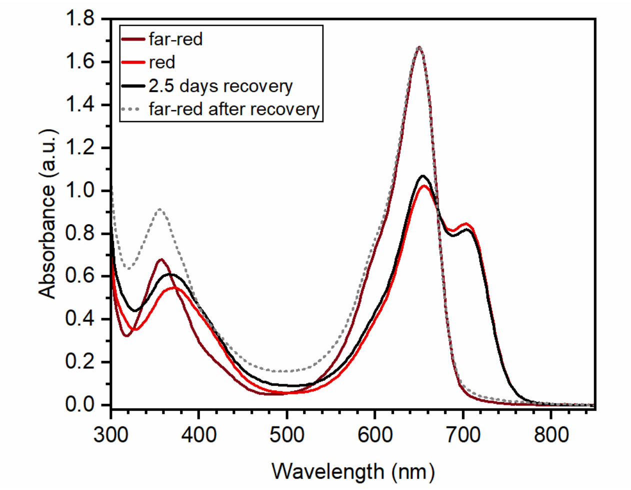

Figure S2. Dark recovery of Cph1-ST. Absorbance spectra of Cph1-ST at 22 °C after pre-illumination with far-red light (0.54 mW/cm², solid dark red line), following saturating exposure to red light (0.07 mW/cm², solid red line) and after recovery for 2.5 days in the dark (black line). A final spectrum was recorded after 2.5 days in the dark and saturating exposure to far-red light (dashed grey line). Based on changes of the absorbance at 700 nm, less than 3% of the total protein reversed back to the P_r state after 2.5 days recovery in the dark, n = 1.

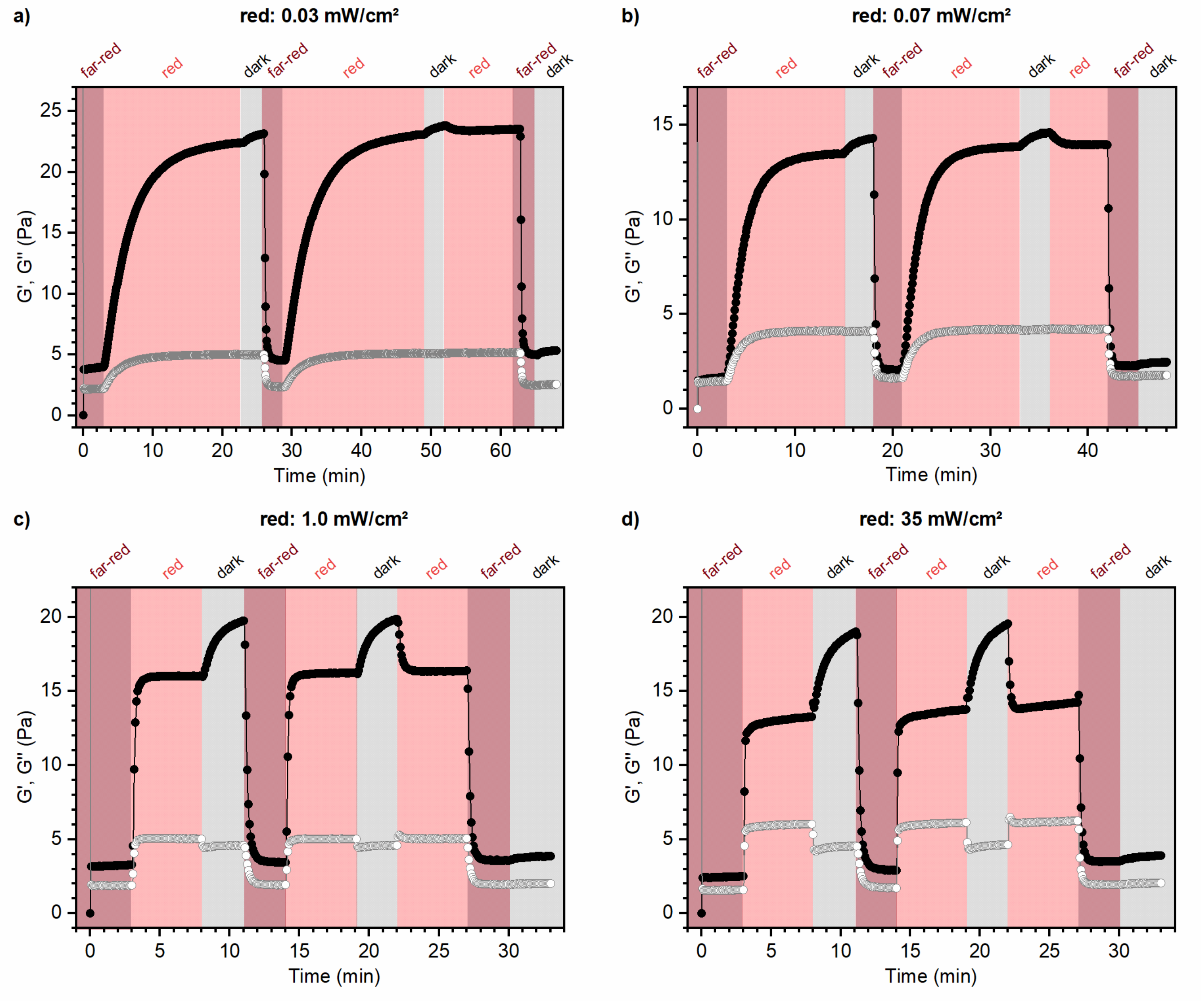

Figure S3. Photorheological characterisation of Cph1-based hydrogels. Exemplary datasets for Cph1 hydrogels from the same batch. During the measurement, hydrogels were illuminated with far-red light (shaded in dark red), red light (shaded in red) or kept in the dark (shaded in grey). While the far-red light intensity was kept constant at 0.54 mW/cm², different red light intensities a) 0.03 mW/cm², b) 0.07 mW/cm², c) 1.0 mW/cm², d) 35 mW/cm² were applied. Shown is one representative hydrogel for each red light intensity, n = 1. Black filled circles represent G' and open grey circles G''. It should be noted that there were substantial batch to batch variations in the absolute values of G' and G'', but the trends were consistent across bathes. To overcome the differences from one batch to the next, the G' data was normalized setting the minimum value of G' under far-red light to zero and the maximum value for G' in the dark-adapted state to 100.

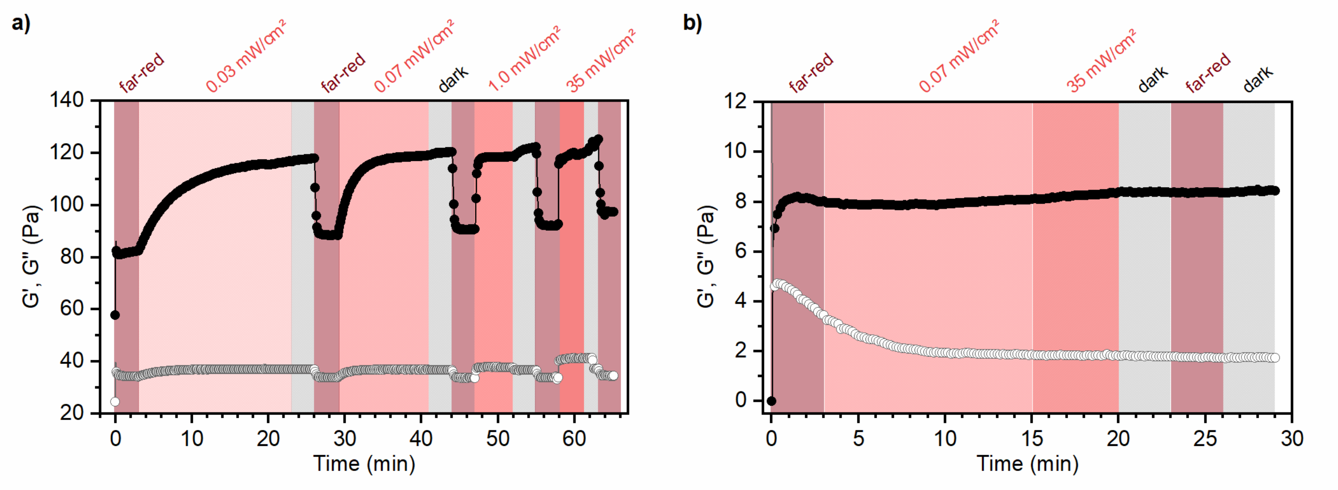

Figure S4. Characterisation of Cph1-based hydrogels. a) Cph1-based hydrogel which was illuminated with red light at intensities of 0.03, 0.07, 1.0 and 35 mW/cm² followed by a dark phase gave rise to identical final G' values. b) AAA-BBB hydrogel showing no significant response to red light at intensities of 0.07 and 35 mW/cm² or far-red light at 0.54 mW/cm². Illumination with far-red is highlighted in dark red, with red in light red and no illumination/ dark in grey. Full squares represent G' and open squares G'', n = 1 for each experiment.

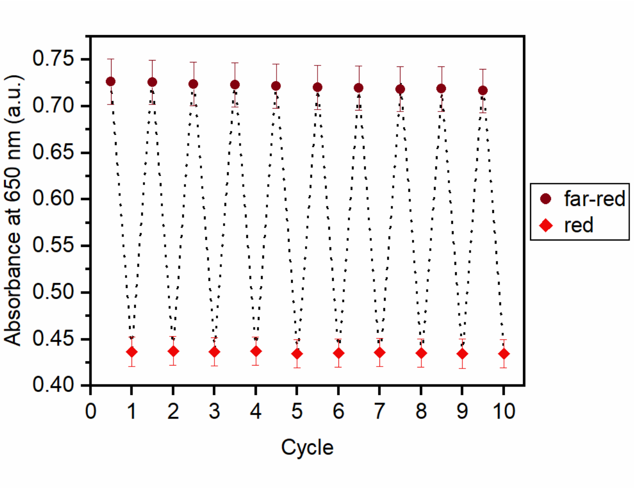

Figure S5. Photocycling of Cph1-ST. The absorbance at 650 nm alternated between high and low values upon saturating illumination with far-red (730 nm, 0.54 mW/cm²) and red light (625 nm, 0.07 mW/cm²), respectively. Cph1 saturated with far-red light is shown as dark red circles, while red light exposed Cph1 is displayed as red squares. Data representing mean with corresponding standard deviation, n = 3.

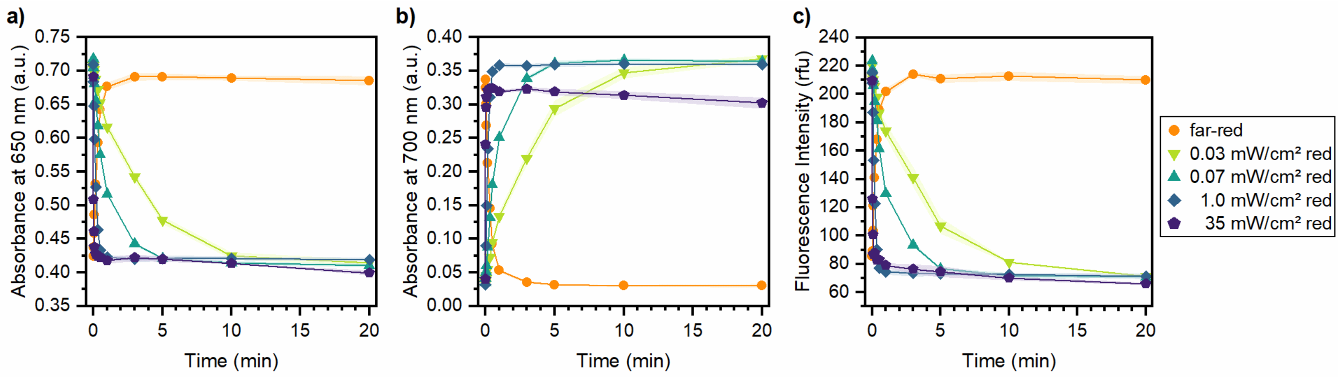

Figure S6. Change in Cph1-ST absorbance at 650 nm (A), 700 nm (b) and fluorescence (c) over time. Cph1 was pre-saturated with far-red light (0.54 mW/cm²), then exposed to red light at intensities of 0.03, 0.07, 1.0 and 35 mW/cm² (light green, blue-green, dark blue and purple line, respectively) to follow $P_r \rightarrow P_{fr}$ photoconversion. $P_{fr} \rightarrow P_r$ reversion (orange line) was followed after saturating illumination with red light (0.07 mW/cm²) and exposure to far-red light (0.54 mW/cm²). Shown is the mean with corresponding standard deviation, n = 3.

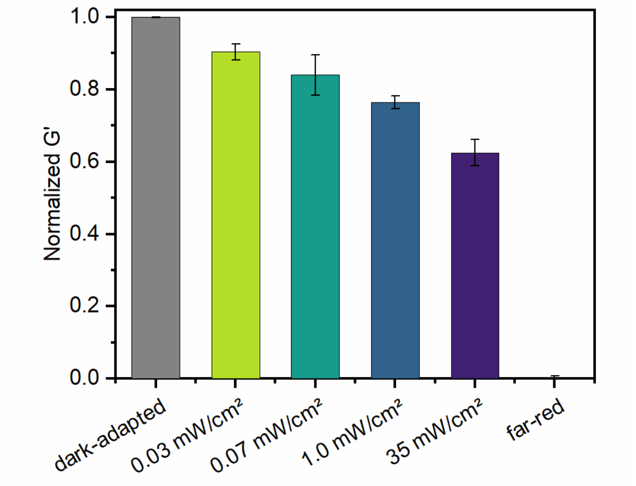

Figure S7. Influence of red light intensity on final hydrogel stiffness. In the dark, after red light illumination ceased, hydrogels show the highest stiffness (grey). As higher the red light intensity, as lower the stiffness (0.03, 0.07, 1.0, 35 mW/cm² in light green, green-blue, dark blue and purple, respectively). After illumination with far-red light, hydrogels show the softest state (orange). Shown is the normalized mean with corresponding standard deviation, n = 3.

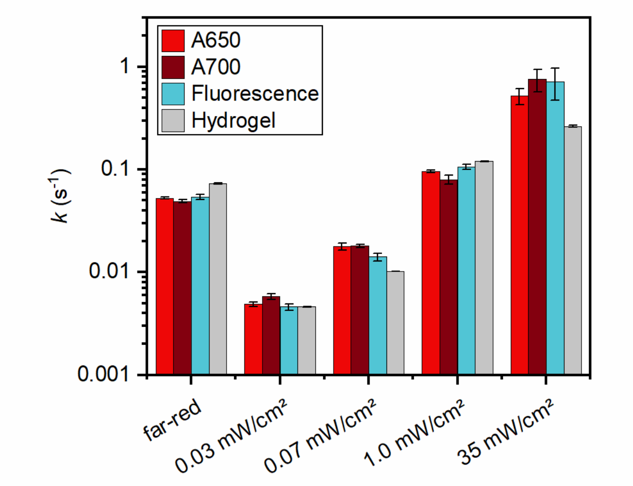

Figure S8. Comparison of rate constant *k*. A higher red light intensity accelerates the rate at which the PSS is approached. Rate constants in solution (determined from the absorbance at 650 nm (A650, light red bars), 700 nm (A700, dark red bars) and the P_r state fluorescence of Cph1 (Fluorescence, blue bars)) and in the hydrogel (Hydrogel, grey bars) are comparable. Data represents mean with corresponding standard deviation, n = 3.

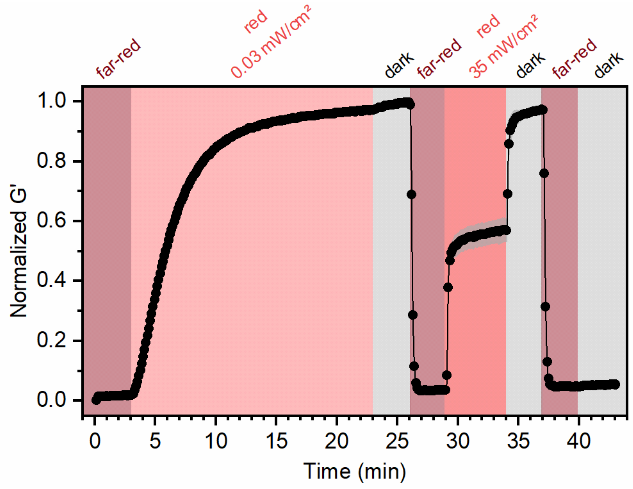

Figure S9. Photorheological characterisation of Cph1-based hydrogels at 1 rad/s angular frequency. During the measurement, hydrogels were illuminated with far-red light (shaded in dark red), red light (shaded in red) or kept in the dark (shaded in grey). While the far-red light intensity was kept constant at 0.54 mW/cm², red light intensity was set to 0.03 mW/cm² (3-23 min) and 35 mW/cm² (29-34 min). Shown is the normalized mean of the storage modulus G' with corresponding standard deviation, n = 3.

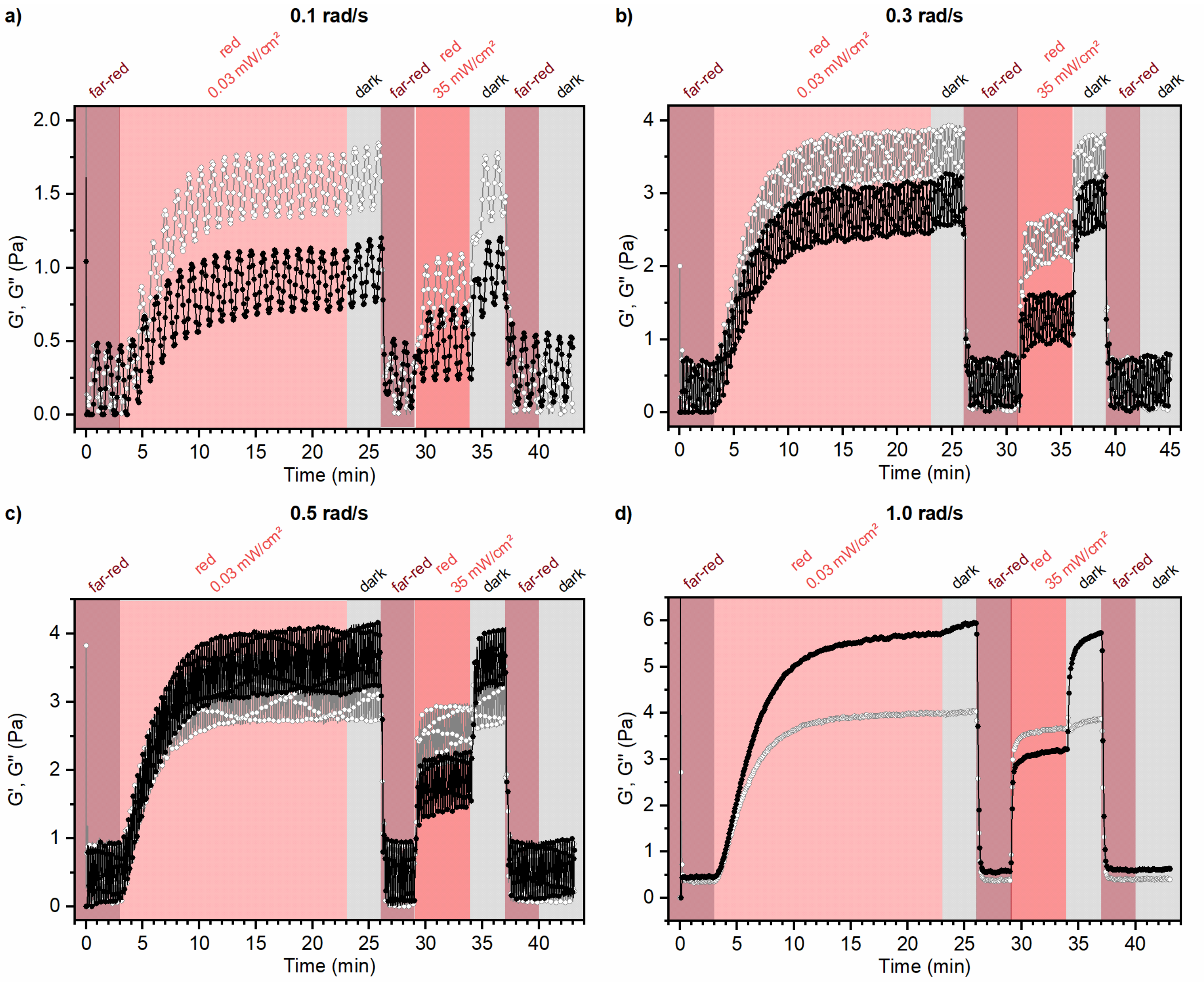

Figure S10. Photorheological characterisation of Cph1-based hydrogels at a) 0.1 rad/s, b) 0.3 rad/s, c) 0.5 rad/s and d) 1.0 rad/s angular frequency. During the measurement, hydrogels were illuminated with far-red light (shaded in dark red), red light (shaded in red) or kept in the dark (shaded in grey). While the far-red light intensity was kept constant at 0.54 mW/cm², red light intensity was set to 0.03 mW/cm² (3-23 min) and 35 mW/cm² (a,c,d: 29-34 min, b: 31-36 min). The storage modulus G' is represented as black filled circles, the loss modulus G'' as grey open circles, n = 1.

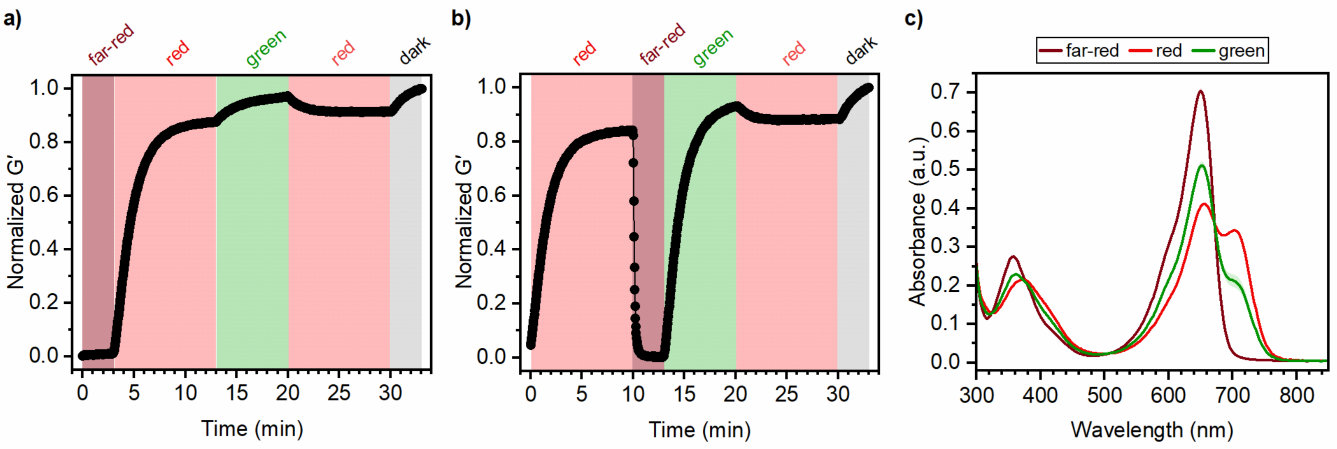

Figure S11. Influence of green light on Cph1-based hydrogels. a) Green light after red light illumination shows a small increase in hydrogel stiffness comparable to the dark-adapted state. b) Green light after far-red light exposure shows a larger increase in hydrogel stiffness, probably due to red light contamination of the green light source. Intensities for far-red, red and green were kept constant at 0.54 mW/cm², 0.07 mW/cm² and 0.8 mW/cm², respectively. Illumination with far-red is highlighted in dark red, with red in light red, with green in green and no illumination/ dark in grey. Shown are the normalized values of G', n = 1. c) Absorbance spectra of Cph1-ST after saturation with far-red light (dark red line), red light (red line) and green light (green line). Intensities are as stated in a) and b). Shown is the mean ± S.D., n = 3.

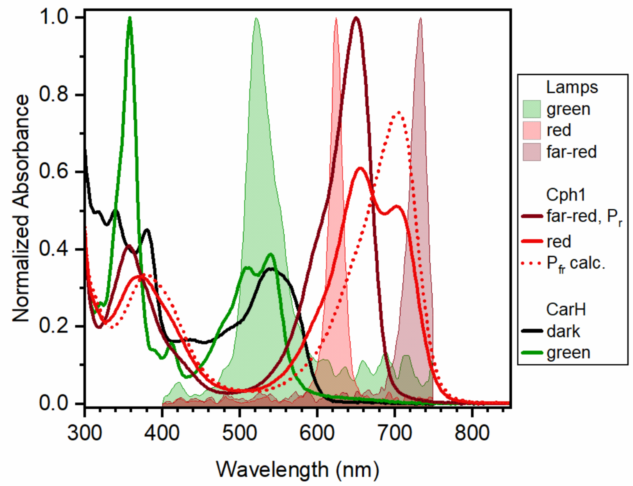

Figure S12. Spectral overlap of used light sources, Cph1 absorbance in the P_r state (dark red solid line), after exposure to red light (red solid line) and in the calculated P_{fr} state (dashed red line) and CarH absorbance in the dark (black solid line) and after green light illumination (green solid line). The far-red light source shows a maximum at 730 nm, the red light source one at 625 nm and the green light source shows a broader spectrum with peak absorbance at 520 nm but residual impurities in the red/ far-red wavelength range.

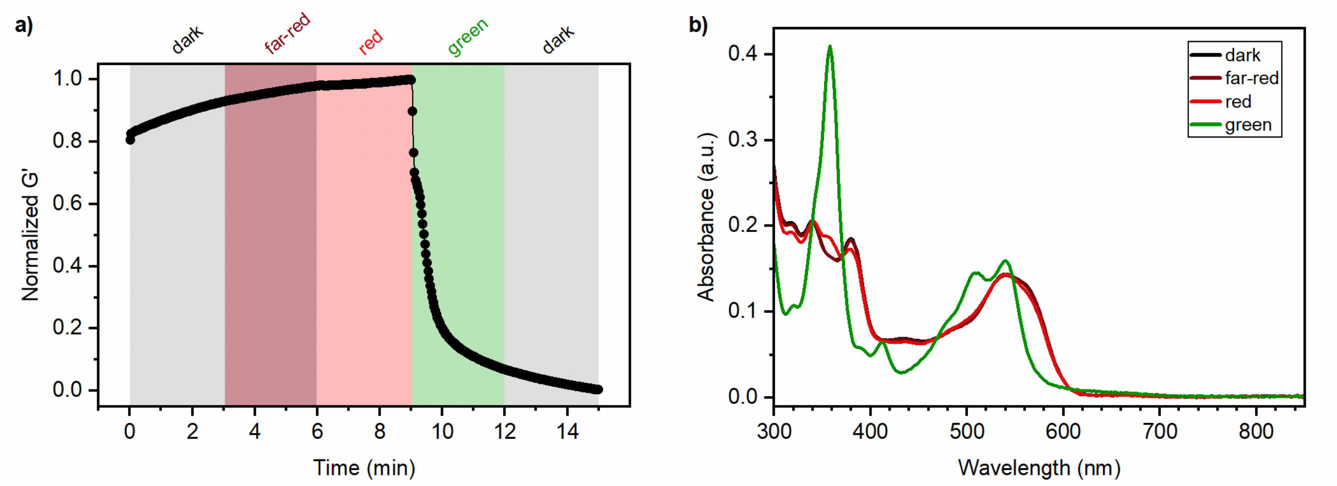

Figure S13. Influence of far-red and red light on CarH. a) CarH-based hydrogels illuminated with far-red (0.54 mW/cm²) and red light (0.07 mW/cm²) show no influence on material properties. Just green light (0.8 mW/cm²) exposure triggers a rapid gel-sol transition. Illumination with far-red is highlighted in dark red, with red in light red, with green in green and no illumination/ dark in grey. Shown are the normalized values of G', n = 1. b) Absorbance spectra of CarH in the dark (black line), under far-red (dark red line), red (red line) and green light (green line) confirm that only green light triggers CarH activity. Shown are mean values ± S.D., n = 3.

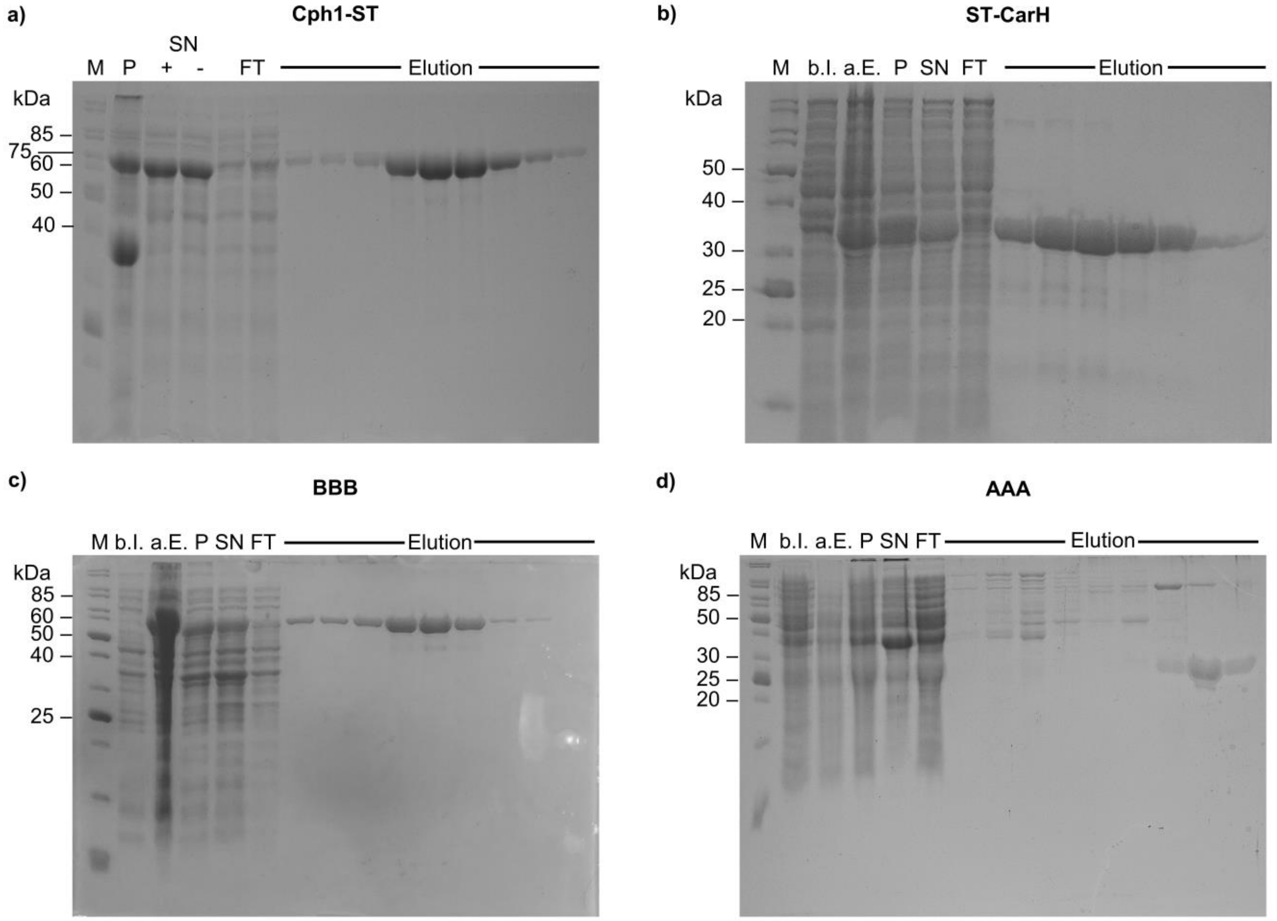

Figure S14. SDS-PAGE after protein purification. a) Cph1-SpyTag (MW: 68.2 kDa), b) SpyTag-CarH (MW: 32.2 kDa), c) BBB (MW: 54.9 kDa) and d) AAA (MW: 20.8 kDa). M: marker, b.I.: before induction, a.E.: after expression, P: pellet after clearance, SN: supernatant after clearance, FT: flow-through after loading onto the column, +: with PCB, -: without PCB. Only elution fractions with high yield and minor impurities were pooled.
